# Strategy‐Specific Effects of Low‐Voltage Area–Targeted Ablation Added to Pulmonary Vein Isolation: A Meta‐Analysis of Randomized Controlled Trials

**DOI:** 10.1002/joa3.70430

**Published:** 2026-07-26

**Authors:** Akihiro Sunaga, Daisuke Sakamoto, Daisaku Nakatani, Katsuki Okada, Hirota Kida, Yuki Matsuoka, Hideaki Hasegawa, Tetsuhisa Kitamura, Masaharu Masuda, Yohei Sotomi, Yasushi Sakata

**Affiliations:** ^1^ Department of Cardiovascular Medicine The University of Osaka Graduate School of Medicine Suita Japan; ^2^ Department of Social and Environmental Medicine The University of Osaka Graduate School of Medicine Suita Japan; ^3^ Cardiovascular Center Kansai Rosai Hospital Amagasaki Japan

**Keywords:** atrial fibrillation, catheter ablation, low‐voltage area, meta‐analysis, substrate modification

## Abstract

**Background:**

Left atrial low‐voltage areas (LVAs) represent an arrhythmogenic substrate in atrial fibrillation (AF). LVA‐targeted ablation is increasingly used as an adjunct to pulmonary vein isolation (PVI), yet outcomes vary across randomized trials, potentially due to differences in ablation strategy. We performed a meta‐analysis to evaluate the effectiveness of LVA‐targeted ablation added to PVI and to assess whether treatment effects differ between LVA isolation and LVA homogenization strategies.

**Methods:**

Following PRISMA, we systematically searched PubMed and CENTRAL for randomized controlled trials (RCTs). Eligible studies enrolled patients with any AF type undergoing voltage mapping and compared PVI plus LVA‐targeted ablation (PVI + LVA) with PVI alone. The primary outcome was AF/atrial tachyarrhythmia recurrence during follow‐up, as defined in each trial. Odds ratios (ORs) with 95% confidence intervals (CIs) were pooled using a random‐effects model. Subgroup analyses were performed according to the predominant LVA strategy (isolation vs. homogenization).

**Results:**

Six RCTs involving 1565 patients (PVI + LVA, *n* = 780; PVI alone, *n* = 785) were included. Overall, PVI + LVA reduced recurrence compared with PVI alone (OR 0.72; 95% CI 0.54–0.96). In strategy‐specific analyses, LVA isolation was associated with a significant reduction in recurrence (OR 0.57; 95% CI 0.38–0.87), whereas LVA homogenization showed a smaller and non‐significant effect (OR 0.85; 95% CI 0.59–1.24). Between‐strategy heterogeneity was substantial (*I* ^2^ = 90.4%).

**Conclusions:**

In RCTs of AF ablation, adding LVA‐targeted ablation to PVI reduced arrhythmia recurrence. The treatment effect appeared more pronounced with LVA isolation than with homogenization; however, these strategy‐specific findings should be considered exploratory and require confirmation in future randomized studies.

## Graphical abstract

Adding LVA‐targeted ablation to PVI was associated with a reduction in atrial tachyarrhythmia recurrence compared with PVI alone. The treatment effect appeared more pronounced in trials employing LVA isolation than in those using homogenization. AF, atrial fibrillation; LVA, low‐voltage area; PVI, pulmonary vein isolation.

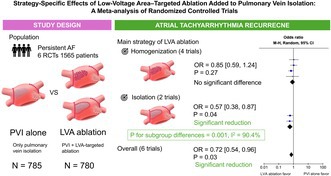

**Abbreviations**
AF: atrial fibrillation
AT: atrial tachycardia
CI: confidence interval
LVA: low‐voltage area
OR: odds ratio
PVI: pulmonary vein isolation
RCT: randomized controlled trial

## 1 Introduction

Pulmonary vein isolation (PVI) is the cornerstone of catheter ablation for atrial fibrillation (AF) [1, 2]. However, arrhythmia recurrence remains a clinically relevant problem after ablation, and atrial substrate abnormalities beyond the pulmonary veins are thought to contribute to AF maintenance and recurrence [3, 4]. Among these, low‐voltage areas (LVAs) identified by electroanatomic voltage mapping are considered markers of atrial fibrosis and an arrhythmogenic substrate associated with adverse rhythm outcomes [5, 6].

Accordingly, LVA‐targeted ablation has been proposed as an adjunctive strategy to PVI [7]. Several randomized controlled trials (RCTs) have evaluated the efficacy of combining PVI with LVA ablation compared with PVI alone, but the results have been inconsistent [8, 9, 10, 11, 12, 13]. One potential explanation for this variability is heterogeneity in LVA ablation strategies. Broadly, two distinct approaches have been employed: LVA isolation, which aims to electrically isolate LVAs by encircling lesions, and LVA homogenization, an ablation approach aimed at eliminating local abnormal electrograms within LVAs.

Whether these different LVA‐targeted strategies confer comparable clinical benefit remains uncertain. Importantly, most individual RCTs were not designed to directly compare isolation and homogenization, and sample sizes were insufficient to draw definitive conclusions. In addition, prior meta‐analyses have included studies with heterogeneous control strategies, not limited to PVI alone, which may have confounded the estimation of the incremental benefit of LVA‐targeted ablation [14, 15]. Therefore, a comprehensive synthesis of available randomized evidence with attention to strategy‐specific effects is warranted.

The present meta‐analysis was conducted to evaluate the overall effectiveness of LVA‐targeted ablation added to PVI compared with PVI alone and, critically, to assess whether treatment effects differ between LVA isolation and LVA homogenization strategies.

## 2 Methods

### 2.1 Search Strategy and Study Selection

This systematic review and meta‐analysis was conducted in accordance with the Preferred Reporting Items for Systematic Reviews and Meta‐Analyses (PRISMA) statement. We systematically searched PubMed and the Cochrane Central Register of Controlled Trials (CENTRAL) from inception through November 5, 2025. Search terms included concepts related to atrial fibrillation, pulmonary vein isolation, low‐voltage areas/voltage mapping, catheter ablation, and randomized trials. The detailed search strategy is provided in the Supporting Information. No language restrictions were applied.

### 2.2 Eligibility Criteria

Studies were eligible if they were randomized controlled trials enrolling patients with any type of atrial fibrillation, in which electroanatomic voltage mapping was used to identify left atrial low‐voltage areas (LVAs), and the intervention arm performed LVA‐targeted ablation in addition to pulmonary vein isolation (PVI) (PVI + LVA group) while the control arm underwent PVI alone without any additional left atrial ablation beyond PVI (PVI alone group). Eligible trials were required to report atrial fibrillation/atrial tachyarrhythmia recurrence beyond the blanking period, as defined by each study. We excluded nonrandomized or observational studies, single‐arm trials, and trials in which the control group underwent any left atrial ablation other than PVI.

### 2.3 Classification of LVA Ablation Strategy

Trials were categorized according to the predominant LVA ablation strategy. Studies were classified as LVA isolation if the protocol primarily involved encircling LVAs with confirmation of electrical isolation (entrance and/or exit block or electrical dissociation). Studies were classified as LVA homogenization if the protocol primarily aimed to eliminate abnormal local electrograms within LVAs through substrate modification without systematic isolation. When localized homogenization for small LVAs was permitted in protocols with isolation as the primary strategy, and when posterior wall isolation was permitted for LVAs on the posterior wall in protocols with homogenization as the primary strategy, trials were classified according to the prespecified predominant strategy, such that the former was categorized as isolation and the latter as homogenization.

### 2.4 Outcome Measures

The primary outcome was AF/atrial tachyarrhythmia recurrence beyond the blanking period, as defined in each trial. Subgroup analyses examined strategy‐specific effects according to LVA ablation approach (isolation vs. homogenization).

### 2.5 Data Extraction

Two investigators independently extracted data (A.S. and D.S.) using a standardized form. Extracted variables included the year of publication, study design, AF type, voltage mapping criteria, ablation strategy, follow‐up duration, and the number of recurrent atrial tachyarrhythmia events in each treatment arm.

### 2.6 Risk of Bias Assessment

Risk of bias was assessed independently by two reviewers using the Cochrane Risk of Bias 2 tool for randomized trials, evaluating bias arising from the randomization process, deviations from intended interventions, missing outcome data, outcome measurement, and selection of the reported result. Disagreements were resolved by consensus, and trial registries were consulted to evaluate selective reporting.

### 2.7 Statistical Analysis

The primary effect measure was the odds ratio (OR) with 95% confidence intervals (CIs) for atrial tachyarrhythmia recurrence. For each trial, we extracted the number of patients with recurrent arrhythmia and the total number of patients in each treatment arm. Pooled estimates were calculated using a Mantel–Haenszel random‐effects model. The Hartung–Knapp–Sidik–Jonkman method was used to derive 95% CIs for the pooled effect, and between‐study variance (τ^2^) was estimated using the DerSimonian–Laird method. Statistical heterogeneity was assessed using Cochran's Q (χ^2^ test) and quantified with the *I* ^2^ statistic. Prespecified subgroup analyses were performed according to the predominant LVA ablation strategy (LVA isolation vs. LVA homogenization), and subgroup differences were evaluated using a χ^2^ test for interaction. Two‐sided *p* values < 0.05 were considered statistically significant. All analyses were conducted using RevMan Web (Cochrane).

## 3 Results

### 3.1 Study Selection

**FIGURE 1:**
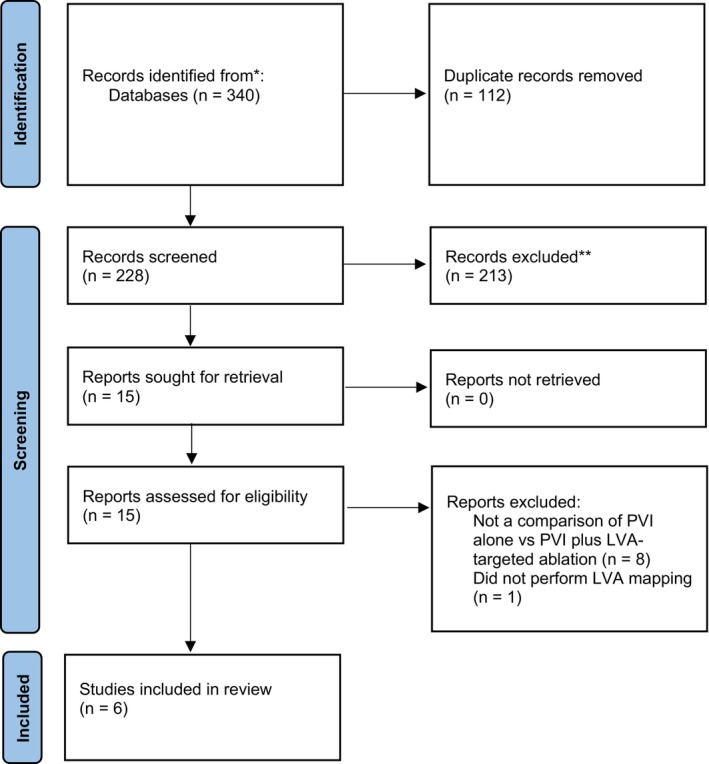
The preferred reporting items for systematic reviews and meta‐analyses (PRISMA) flow diagram of included studies. Records were identified through database searches (PubMed and CENTRAL; *n* = 340). After removal of duplicates (*n* = 112), *n* = 228 records were screened by title/abstract. Fifteen reports were assessed for eligibility, and nine were excluded (control group not PVI alone without additional left atrial ablation, *n* = 8; no LVA mapping performed, *n* = 1). Finally, six randomized controlled trials were included in the qualitative and quantitative synthesis (meta‐analysis). LVA, low‐voltage area; PVI, pulmonary vein isolation.

Of 228 potential articles screened, 6 randomized controlled trials including 1565 patients (PVI + LVA group, *n* = 780; PVI alone group, *n* = 785) were included in the meta‐analysis. (Figure 1) Table 1 summarizes the characteristics of included studies. Two of the six trials enrolled patients with paroxysmal AF, comprising a total of 500 participants (32%). The remaining four trials included patients with persistent AF. Among the six trials, four had a follow‐up duration of 12 months, whereas the remaining two had follow‐up periods of 18 months and 23 months, respectively. Table 2 summarizes the methodology of voltage mapping. In five of the six trials, low‐voltage areas were defined as regions with bipolar voltage amplitudes ≤ 0.5 mV, whereas the remaining trial used a cutoff of ≤ 0.4 mV.

**TABLE 1:** Characteristics of included studies.

Study (author)	LVA ablation strategy (endpoint)	AF type	Energy source	Follow‐up	Primary endpoint
SUPPRESS‐AF (Masuda M, et al.)	Homogenization (Visitag Surpoint of ≥ 350 with an interlesion distance of < 6 mm)	PeAF	Radiofrequency	24‐h Holter at 6 and 12 months; Portable ECG during months 6–12	The recurrence of AF/AT > 30 s without antiarrhythmic drug use during the 1‐year follow‐up
ERASE‐AF (Huo Y, et al.)	Isolation (bidirectional conduction block confirmed)	PeAF	Radiofrequency	7‐day ECG recordings at 3, 6, 12 months. Some patients with ICM implantation	The recurrence of AF/AT > 30 s during the 1‐year follow‐up
SCAR‐AF (Lepillier A, et al.)	Homogenization (elimination of all low‐amplitude and fractionated electrograms)	PeAF	Radiofrequency	24‐h Holter at 3, 6, 12, 18 months	The recurrence of AF/AT > 30 s during the 18‐month follow‐up
STABLE‐SR II (Yang G, et al.)	Homogenization (reduction of local electrogram amplitude to < 0.1 mV)	PeAF	Radiofrequency	24‐h Holter at 3 months and 6 months; 7‐day Holter at 12 months; thereafter 24‐h Holter every 6 months until the trial was closed	The recurrence of AF/AT > 30 s during the 1‐year follow‐up
VOLCANO (Masuda M, et al.)	Homogenization (> 50% reduction in electrogram voltage)	PAF	Radiofrequency or cryothermy	12‐lead ECG at 1, 3, 6, 9, 12 months; 24‐h Holter at 6 and 12 months	The recurrence of AF/AT > 30 s without antiarrhythmic drug use during the 1‐year follow‐up
STABLE‐SR III (Chen H, et al.)	Isolation (bidirectional conduction block confirmed)	PAF	Radiofrequency	24‐h Holter at 3 and 6 months; 7‐day Holter at 12 months; thereafter 24‐h Holter every 6 months until the trial was closed	The recurrence of AF/AT > 30 s without antiarrhythmic drug use during the observation period. Median follow‐up was 23 months

Abbreviations: AF, atrial fibrillation; AT, atrial tachycardia; ECG, electrocardiogram; ICM, implantable cardiac monitor; LA, left atrial; LVA, low‐voltage area; PAF, paroxysmal atrial fibrillation; PeAF, persistent atrial fibrillation.

**TABLE 2:** Voltage mapping methodology and definitions of low‐voltage areas in the included trials.

Study (author)	3D mapping system	Mapping catheter	Mapping rhythm	LVA definition
SUPPRESS‐AF (Masuda M, et al.)	CARTO 3	LASSO or PentaRay	High right atrial pacing (100 ppm)	Bipolar voltage < 0.5 mV. “LVA‐positive” if total LVA area > 5 cm^2^
ERASE‐AF (Huo Y, et al.)	CARTO 3 or EnSite	LASSO or Reflexion	Sinus rhythm	Bipolar voltage < 0.5 mV
SCAR‐AF (Lepillier A, et al.)	CARTO 3 or RHYTHMIA or EnSite	Multipolar catheter	Sinus rhythm	Bipolar voltage < 0.5 mV
STABLE‐SR II (Yang G, et al.)	CARTO 3	LASSO or PentaRay	Sinus rhythm	Bipolar voltage < 0.1 mV as dense scar; 0.1–0.4 mV as LVA; 0.4–1.3 mV as transitional zone. Patients with normal LA substrate were defined as those who had LVA/LA < 1% and with no complex electrogram in TZ. The remaining patients were classified into the abnormal substrate group
VOLCANO (Masuda M, et al.)	CARTO 3 or EnSite	LASSO or Inquiry Optima	Sinus rhythm	Bipolar voltage < 0.5 mV. “LVA‐positive” if total LVA area > 5 cm^2^
STABLE‐SR III (Chen H, et al.)	CARTO 3	SmartTouch catheter	Sinus rhythm	Bipolar voltage < 0.5 mV in > 3 adjacent points with space difference of 0.5 cm

*Note:* CARTO 3, LASSO, PentaRay, and SmartTouch are products of Biosense Webster (Irvine, CA, USA); RHYTHMIA is a product of Boston Scientific (Marlborough, MA, USA); EnSite, Reflexion, and Inquiry Optima are products of Abbott (St. Paul, MN, USA).

Abbreviations: 3D, three‐dimensional; LA, left atrial; LVA, low‐voltage area; ppm, paced per minute; TZ, transitional zone.

Table 3 summarizes the baseline characteristics of included patients. Out of 1565 patients, 674 were female (43%). The mean age of each trial ranged from 60 to 75 years. In the SUPPRESS‐AF, SCAR‐AF, and VOLCANO trials, only patients with identified LVAs were randomized; therefore, the prevalence of LVAs among randomized participants was 100%. However, among patients who underwent voltage mapping, LVAs were identified in 25.3% in SUPPRESS‐AF (339/1341), 70.0% in SCAR‐AF (148/211), and 15.6% in VOLCANO (62/398).

**TABLE 3:** Baseline characteristics.

	SUPPRESS‐AF		ERASE‐AF		STABLE‐SR‐II		SCAR‐AF		VOLCANO		STABLE‐SR‐III	
	LVA ablation	PVI alone	LVA ablation	PVI alone	LVA ablation	PVI alone	LVA ablation	PVI alone	LVA ablation	PVI alone	LVA ablation	PVI alone
Patients	170	171	161	163	134	142	76	72	30	32	219	219
Age, years	73.6 ± 6.8	74.7 ± 6.1	65 ± 10	66 ± 10	60.6 ± 9.4	60.4 ± 9.6	65.4 ± 8.9	66.9 ± 7.9	75.3 ± 7.2	74.7 ± 8.0	70.2 ± 4.7	70.7 ± 4.1
Female, *n* (%)	85 (50)	82 (48)	49 (30)	59 (36)	46 (34)	43 (30)	22 (29)	25 (35)	21 (70)	23 (72)	108 (49)	111 (51)
LVA, *n* (%)	170 (100)	171 (100)	54 (34)	64 (39)	71 (55)	62 (46)	76 (100)	72 (100)	30 (100)	32 (100)	88 (40)	93 (43)
AF history, months	6 (1, 11)	6 (1, 10)	31 (8–77)	31 (12–77)	N.R.	N.R.	N.R.	N.R.	4 (2, 14)	4 (2, 23)	24 (6, 48)	14 (4, 48)
HT, *n* (%)	117 (68)	125 (74)	130 (81)	137 (84)	65 (49)	58 (41)	49 (65)	45 (63)	20 (67)	16 (50)	130 (60)	142 (65)
DM, *n* (%)	42 (25)	35 (21)	40 (25)	40 (25)	11 (8)	14 (11)	14 (18)	19 (26)	10 (33)	6 (19)	32 (15)	43 (20)
HF, *n* (%)	54 (32)	49 (29)	32 (20)	42 (28)	4 (3)	1 (1)	N.R.	N.R.	5 (17)	6 (19)	2 (1)	1 (1)
Stroke, *n* (%)	15 (9)	19 (11)	12 (7)	15 (9)	5 (4)	4 (3)	7 (9)	0 (0)	N.R.	N.R.	17 (8)	21 (10)
CHA2DS2‐VASc	3.4 ± 1.3	3.5 ± 1.5	3 (2, 4)	3 (2, 4)	^a^	^a^	2.5 ± 1.7	2.4 ± 1.4	3.6 ± 1.2	3.3 ± 1.3	2.3 ± 0.8	2.5 ± 1.0
LVEF, %	55.8 ± 10.7	57.4 ± 10.4	53 ± 12	54 ± 11	61.3 ± 9.2	62.1 ± 6.8	50.2 ± 14	51.3 ± 10.7	64 ± 14	65 ± 10	62.4 ± 5.3	62.4 ± 5.4
LAD, mm	44.1 ± 5.4	43.6 ± 5.5	45 ± 7	45 ± 6	41.4 ± 5.9	42.5 ± 5.3	N.R.	N.R.	40 ± 6	38 ± 5	38.8 ± 5.4	38.8 ± 5.4

Abbreviations: AF, atrial fibrillation; DM, diabetes mellitus; HF, heart failure; HT, hypertension; LAD, left atrial diameter; LVA, low‐voltage area; LVEF, left ventricular ejection fraction; N.R., not reported; PVI, pulmonary vein isolation.

^a^ Patients with a CHA_2_DS_2_‐VASc score ≥ 3 numbered 23 (17.1%) in the LVA ablation group and 19 (13.4%) in the PVI alone group.

**FIGURE 2:**
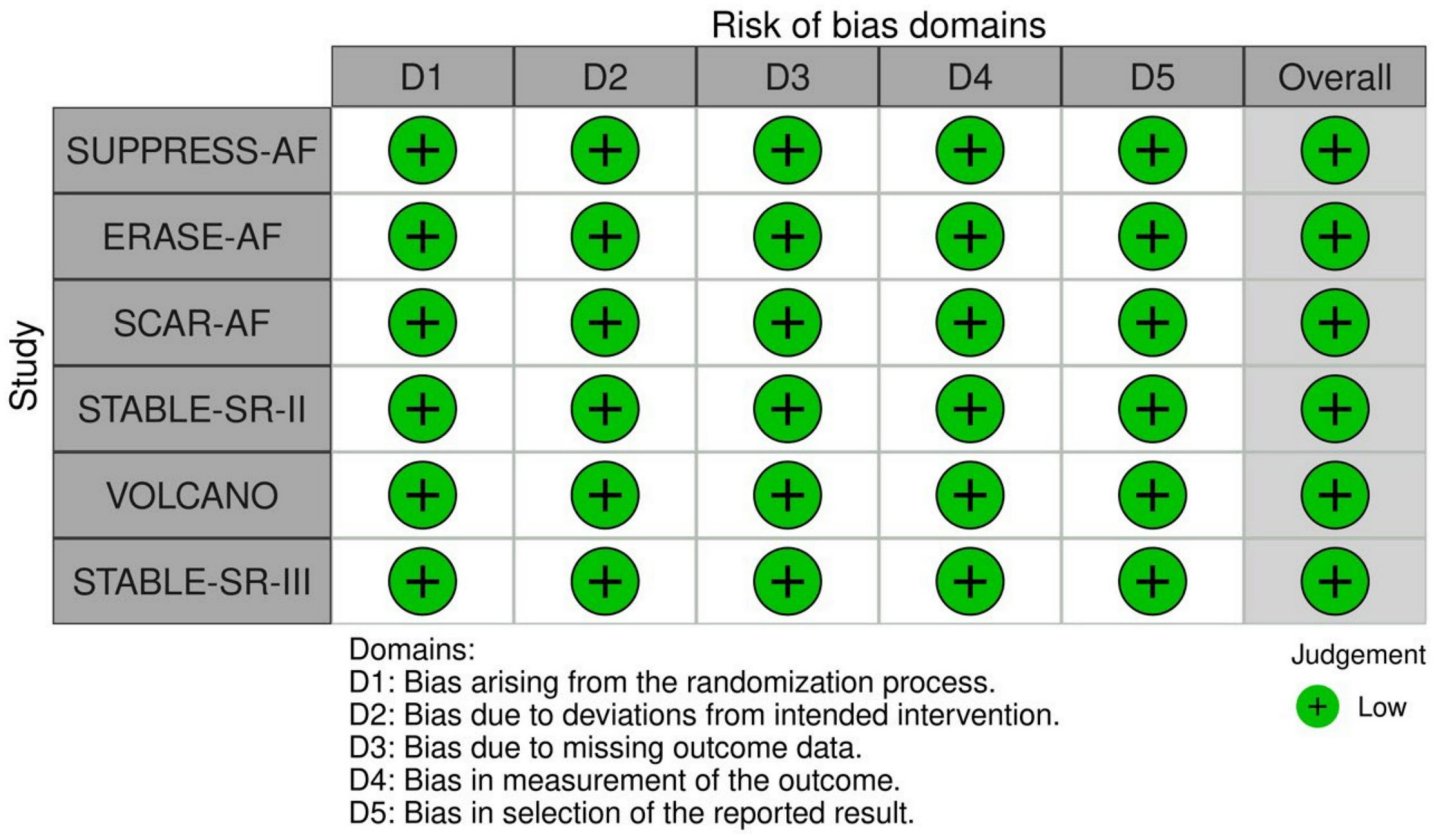
Risk of bias assessment of included randomized controlled trials. Risk of bias was evaluated using the Cochrane Risk of Bias 2 (RoB 2) tool across five domains: Bias arising from the randomization process (D1), bias due to deviations from intended interventions (D2), bias due to missing outcome data (D3), bias in measurement of the outcome (D4), and bias in selection of the reported result (D5). Each domain and the overall risk of bias were judged as low risk for all included trials.

Figure 2 shows the risk of bias assessment, which was judged to be low in all studies. Funnel plots did not suggest marked asymmetry; however, interpretation is limited because fewer than 10 trials were included (Figure S1).

### 3.2 Outcome

**FIGURE 3:**
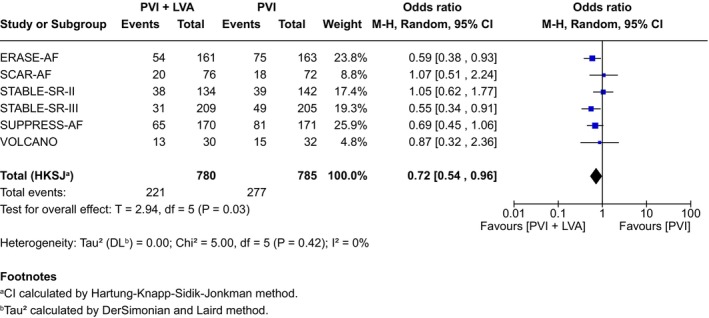
Effect of adding LVA‐targeted ablation to pulmonary vein isolation on atrial fibrillation recurrence. Forest plot showing odds ratios (ORs) with 95% confidence intervals (CIs) for atrial fibrillation/atrial tachyarrhythmia recurrence comparing PVI plus LVA‐targeted ablation (experimental) versus PVI alone (control). Pooled estimates were calculated using a random‐effects Mantel–Haenszel model with Hartung–Knapp–Sidik–Jonkman adjustment. Overall, LVA‐targeted ablation was associated with a significantly lower recurrence risk compared with PVI alone (OR 0.72, 95% CI 0.54–0.96). No significant between‐study heterogeneity was observed (*I* ^2^ = 0%). LVA, low‐voltage area; PVI, pulmonary vein isolation.

**FIGURE 4:**
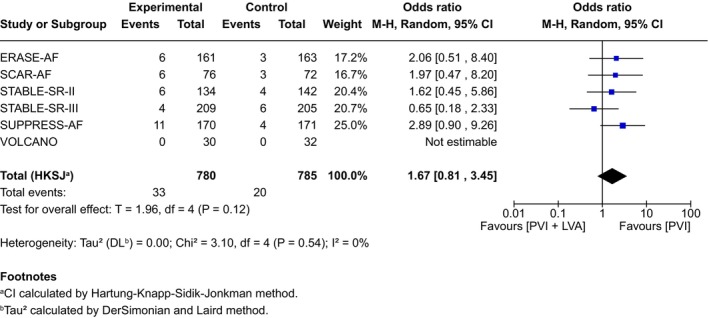
Procedural complications comparing PVI plus LVA‐targeted ablation versus PVI alone. Forest plot showing odds ratios (ORs) with 95% confidence intervals (CIs) for procedure‐related complications comparing PVI plus LVA‐targeted ablation (experimental) with PVI alone (control). Pooled estimates were calculated using a random‐effects Mantel–Haenszel model with Hartung–Knapp–Sidik–Jonkman adjustment. There was no significant difference in complication rates between the two groups (OR 1.67, 95% CI 0.81–3.45; *p* = 0.12), with no evidence of between‐study heterogeneity (*I* ^2^ = 0%). LVA, low‐voltage area; PVI, pulmonary vein isolation.

Overall, LVA‐targeted ablation added to PVI was associated with a significantly lower risk of atrial tachyarrhythmia recurrence compared with PVI alone (OR 0.72, 95% CI 0.54–0.96; *p* = 0.03), with no evidence of heterogeneity across trials (*I* ^2^ = 0%). (Figure 3) Adding LVA ablation to PVI was not associated with a significant difference in complication risk. (OR 1.67, 95% CI 0.81–3.45; *p* = 0.12) (Figure 4) The types of major procedure‐related complications reported in the included trials are summarized in Table S1.

**FIGURE 5:**
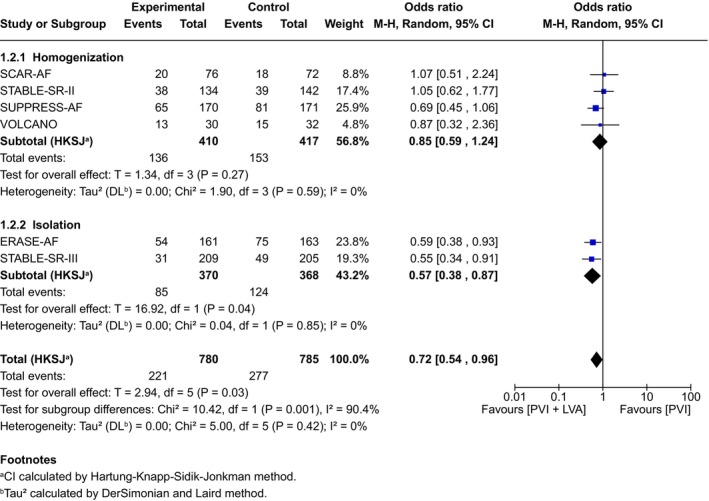
Strategy‐specific effects of LVA‐targeted ablation added to pulmonary vein isolation. Forest plot showing odds ratios (ORs) with 95% confidence intervals (CIs) for atrial fibrillation/atrial tachyarrhythmia recurrence comparing PVI plus LVA‐targeted ablation versus PVI alone, stratified by LVA ablation strategy (homogenization vs. isolation). Pooled estimates were calculated using a random‐effects Mantel–Haenszel model with Hartung–Knapp–Sidik–Jonkman adjustment. LVA isolation was associated with a significant reduction in recurrence, whereas homogenization showed no significant benefit. A significant difference between strategies was observed (test for subgroup differences: *p* = 0.001). LVA, low‐voltage area; PVI, pulmonary vein isolation.

In prespecified subgroup analyses according to the predominant LVA ablation strategy, LVA isolation was associated with a significant reduction in recurrence (OR 0.57, 95% CI 0.38–0.87; *p* = 0.04), whereas LVA homogenization showed a smaller, non‐significant effect (OR 0.85, 95% CI 0.59–1.24; *p* = 0.27). There was strong evidence of a differential treatment effect and heterogeneity between strategies (*p* for subgroup differences = 0.001, *I* ^2^ = 90.4%) (Figure 5).

### 3.3 Leave‐One‐Out Sensitivity Analyses

To evaluate the robustness of the primary analysis, we repeated the meta‐analysis after sequentially excluding each trial (Figure S2). The pooled effect estimate remained broadly consistent across analyses, with summary ORs ranging from 0.67 to 0.77. However, in several analyses, the pooled estimate had a 95% confidence interval that crossed 1.0, and thus the statistical significance of the overall effect was not consistent across all iterations.

Specifically, the reduction in recurrence remained significant after excluding either SCAR‐AF or STABLE‐SR II (OR 0.69 [0.50–0.95] and OR 0.67 [0.50–0.88], respectively). In contrast, when ERASE‐AF, STABLE‐SR III, SUPPRESS‐AF, or VOLCANO was excluded, the pooled OR remained in the same direction but the confidence interval crossed unity (OR 0.77 [0.54–1.10], OR 0.77 [0.55–1.07], OR 0.74 [0.50–1.11], and OR 0.72 [0.50–1.03], respectively), indicating attenuation of the overall statistical significance.

Across all leave‐one‐out analyses, the isolation strategy remained consistently associated with a significant benefit (driven by the ERASE‐AF and/or STABLE‐SR III trials, depending on which study was removed), whereas the homogenization subgroup remained non‐significant in all analyses and, when SUPPRESS‐AF was excluded, shifted toward a null effect (OR 1.02 [0.82–1.27]).

The between‐strategy interaction (test for subgroup differences) was significant in most iterations (typically *p* ≈0.01–0.02) but became non‐significant when excluding ERASE‐AF (*p* = 0.12) or STABLE‐SR III (*p* = 0.16). When SUPPRESS‐AF was excluded, the interaction became markedly larger (χ^2^ = 91.29; *p* < 0.00001), reflecting the homogenization subgroup moving closer to the null while the isolation effect remained favorable.

## 4 Discussion

### 4.1 Main Finding

In this meta‐analysis of randomized controlled trials, adding LVA‐targeted ablation to pulmonary vein isolation was associated with a statistically significant reduction in atrial tachyarrhythmia recurrence. Notably, treatment effects appeared strategy dependent: trials employing an isolation‐based approach demonstrated a substantial reduction in recurrence, whereas trials using homogenization‐based approaches showed a smaller and non‐significant effect. The significant subgroup difference suggests that the manner in which fibrotic substrate is modified—rather than the mere addition of substrate ablation—may be a key determinant of clinical benefit.

### 4.2 Comparison With Prior Meta‐Analyses

An important distinction of the present study from prior meta‐analyses is the restriction of the control arm to PVI alone across all included randomized trials. Previous meta‐analyses of LVA‐ or fibrosis‐guided ablation have included studies with heterogeneous comparator strategies or broader substrate‐modification approaches, which may have confounded estimation of the incremental benefit of LVA‐targeted ablation beyond standard PVI [14, 15, 16]. By focusing on a uniform PVI‐only comparator, our analysis provides a more clinically interpretable estimate of the additive value of LVA modification and allows clearer assessment of strategy‐specific effects.

### 4.3 Why Isolation May Outperform Homogenization

Several mechanistic considerations may explain these strategy‐specific findings. LVA isolation aims to electrically compartmentalize fibrotic regions by creating encircling lesion sets with confirmation of entrance/exit block or electrical dissociation, thereby reducing conduction through slow‐conducting channels and limiting localized reentry within scarred tissue. In addition, because isolation typically encompasses the surrounding border zone, it may also eliminate partially diseased tissue adjacent to LVAs that is not strictly “low‐voltage” yet can still harbor arrhythmogenic drivers; prior mechanistic work has suggested that AF‐sustaining sources may preferentially localize near fibrosis border regions [17].

By contrast, homogenization targets abnormal local electrograms within LVA regions through distributed lesion deployment without a uniform anatomic or electrophysiological endpoint. As a result, its effectiveness may depend strongly on lesion depth, durability, and complete coverage of critical electrogram targets, making it more sensitive to operator technique, mapping resolution, and the spatial distribution of fibrosis. Moreover, rather than uniformly abolishing substrate, extensive but incomplete homogenization can create islands of surviving myocardium and corridors of slow conduction between ablation lesions and anatomic barriers, providing a potential isthmus for macroreentry and thereby promoting iatrogenic atrial tachycardia [18].

Analogous to scar‐related ventricular tachycardia, in which reentry is maintained by surviving myocardial corridors within the scar border zone (“conducting channels”), arrhythmogenic substrate may be localized to discrete channels or border zones rather than uniformly distributed throughout low‐voltage regions [19, 20, 21]. An isolation strategy may therefore be more likely to interrupt these critical pathways in a reproducible manner, whereas homogenization may variably modify them depending on electrogram identification and lesion delivery.

### 4.4 Procedural Standardization and Reproducibility

An additional consideration is procedural safety and feasibility. Homogenization often entails extensive ablation within the atrial myocardium. To mitigate collateral injury, several protocols allowed posterior wall isolation rather than extensive posterior homogenization when LVAs were located adjacent to the esophagus. While these pragmatic modifications are clinically appropriate, they also underscore the complexity of implementing a “pure” homogenization strategy in routine practice. In contrast, isolation‐based protocols typically prespecify a clear endpoint—electrical isolation—which may improve procedural standardization and reproducibility across centers.

### 4.5 Clinical Implication

Clinically, these findings suggest that when LVA‐targeted substrate modification is added to PVI, strategies that prioritize electrical isolation of LVA regions may be more likely to improve rhythm outcomes than extensive homogenization alone. The results also highlight the need for standardized voltage criteria, procedural endpoints, and follow‐up surveillance to ensure reproducible implementation in routine practice.

### 4.6 Limitations

Our meta‐analysis has several limitations. First, the number of trials within each strategy subgroup—particularly for isolation—was small, and subgroup comparisons in meta‐analysis are inherently observational at the trial level. Although the interaction test suggested strategy‐specific differences, these findings should be interpreted as hypothesis generating rather than definitive evidence that one strategy is superior. Furthermore, leave‐one‐out sensitivity analyses demonstrated that the statistical significance of both the overall treatment effect and the between‐strategy interaction was not consistently preserved after exclusion of certain trials, indicating that these findings remain sensitive to individual studies.

Second, classification of LVA ablation strategy was based on the prespecified predominant approach; however, actual procedural details were not completely uniform across trials. Several protocols permitted pragmatic modifications, such as localized homogenization in isolation‐based strategies or posterior wall isolation in homogenization‐based strategies. In addition, detailed procedural information, including the frequency of posterior wall isolation, additional linear ablation, and achievement of complete LVA isolation or homogenization, was not consistently reported across studies. Such overlap and incomplete reporting may have introduced misclassification and diluted true between‐strategy differences.

Third, methodologies used for LVA identification were not fully standardized across trials. Differences in mapping systems, mapping catheters, mapping rhythm, mapping density, and voltage criteria may have influenced the extent and distribution of detected LVAs, thereby affecting both patient selection for LVA‐targeted ablation and the interpretation of strategy‐specific treatment effects.

Fourth, follow‐up duration varied across trials (12–23 months), and we therefore used outcomes reported at each study's follow‐up time point rather than standardizing to a fixed time horizon. This approach improves consistency with the original trial reporting but may contribute to heterogeneity because longer surveillance can increase the likelihood of detecting recurrences.

Fifth, the analysis of procedural safety was based on a relatively small number of events. Although no statistically significant difference in complication rates was observed between groups, the confidence intervals were wide, and the study may have been underpowered to detect modest but clinically relevant differences in procedural risk. Therefore, an increase in procedure‐related complications associated with adjunctive LVA ablation cannot be definitively excluded.

Sixth, two of the included trials (SUPPRESS‐AF and VOLCANO) were conducted by closely related research groups and shared similar approaches to patient selection, voltage mapping, and procedural workflow. Although leave‐one‐out analyses showed a generally consistent direction of effect, the inclusion of studies with potentially overlapping procedural philosophies may limit the independence and generalizability of the available evidence.

Finally, recurrence definitions and monitoring intensity differed among studies, ranging from periodic Holter monitoring to prolonged monitoring, which may have affected the measured recurrence rates and limited direct comparability across trials.

## 5 Conclusion

In this meta‐analysis of randomized trials, adding LVA‐targeted ablation to PVI was associated with a reduction in atrial tachyarrhythmia recurrence compared with PVI alone. The treatment effect appeared more pronounced in trials employing LVA isolation than in those using homogenization; however, these strategy‐specific findings should be considered exploratory and require confirmation in future studies.

## Funding

The authors have nothing to report.

## Conflicts of Interest

The authors declare no conflicts of interest.

## Supporting information


**Figure S1:** Funnel plot for assessment of publication bias.
**Figure S2:** Leave‐one‐out sensitivity analyses of atrial fibrillation recurrence.
**Table S1:** Major procedure‐related complications.

## Data Availability

The data that support the findings of this study are available on request from the corresponding author. The data are not publicly available due to privacy or ethical restrictions.
