# Supplementary material for: Strategy‐Specific Effects of Low‐Voltage Area–Targeted Ablation Added to Pulmonary Vein Isolation: A Meta‐Analysis of Randomized Controlled Trials
Source: J Arrhythm. 2026 Jul 26;42(4):e70430. doi: 10.1002/joa3.70430 (PMC13402018; doi:10.1002/joa3.70430)
Supplement: Supplementary file 1 — Figure S1: Funnel plot for assessment of publication bias. Figure S2: Leave‐one‐out sensitivity analyses of atrial fibrillation recurrence. Table S1: Major procedure‐related complications. [file JOA3-42-e70430-s001.docx]

**Supplementary Material**

**Supplementary Figure 1. Funnel plot for assessment of publication bias.**

Funnel plot of log odds ratios versus standard errors for atrial fibrillation/atrial tachyarrhythmia recurrence comparing PVI plus LVA-targeted ablation with PVI alone. Each point represents an individual randomized controlled trial.

PVI, pulmonary vein isolation; LVA, low-voltage area.

**Supplementary figure 2. Leave-one-out sensitivity analyses of atrial fibrillation recurrence.**

Forest plots showing sensitivity analyses in which each included randomized controlled trial was sequentially excluded to assess the robustness of the pooled effect of PVI plus LVA-targeted ablation versus PVI alone on atrial fibrillation/atrial tachyarrhythmia recurrence. Panels A–F present analyses excluding (A) STABLE-SR II, (B) SCAR-AF, (C) VOLCANO, (D) SUPPRESS-AF, (E) STABLE-SR III, and (F) ERASE-AF, respectively. Pooled odds ratios (ORs) with 95% confidence intervals (CIs) were calculated using a random-effects Mantel–Haenszel model with Hartung–Knapp–Sidik–Jonkman adjustment. Overall estimates remained directionally consistent across analyses, although statistical significance varied depending on the excluded study.

PVI, pulmonary vein isolation; LVA, low-voltage area.

**Supplementary Table. Major procedure-related complications**

|  | SUPPRESS-AF | | ERASE-AF | | STABLE-SR-II | | SCAR-AF | | VOLCANO | | STABLE-SR-III | |
| --- | --- | --- | --- | --- | --- | --- | --- | --- | --- | --- | --- | --- |
|  | LVA ablation | PVI alone | LVA ablation | PVI alone | LVA ablation | PVI alone | LVA ablation | PVI alone | LVA ablation | PVI alone | LVA ablation | PVI alone |
| Patients | 170 | 172* | 161 | 163 | 134 | 142 | 76 | 72 | 30 | 32 | 219 | 219 |
| Tamponade/ effusion, n (%) | 0 (0) | 1 (0.6) | 2 (1.2) | 0 (0) | 0 (0) | 1 (0.7) | 3 (3.9) | 2 (2.6) | 0 (0) | 0 (0) | 0 (0) | 0 (0) |
| Stroke/systemic embolism, n (%) | 0 (0) | 1 (0.6) | 0 (0) | 0 (0) | 1 (0.7) | 0 (0) | 0 (0) | 0 (0) | 0 (0) | 0 (0) | 0 (0) | 0 (0) |
| Esophageal fistula, n (%) | 1 (0.6) | 0 (0) | 0 (0) | 0 (0) | 0 (0) | 0 (0) | 0 (0) | 0 (0) | 0 (0) | 0 (0) | 0 (0) | 0 (0) |
| Death, n (%) | 0 (0) | 0 (0) | 0 (0) | 0 (0) | 0 (0) | 0 (0) | 0 (0) | 0 (0) | 0 (0) | 0 (0) | 0 (0) | 0 (0) |

*In SUPPRESS-AF, the safety analysis set included 172 patients in the PVI-alone group.

LVA, low-voltage area; PVI, pulmonary vein isolation.
